# Tröpfchenexposition bei Tracheotomie: Fallanalyse und Konsequenzen in Bezug auf COVID-19-Patienten

**DOI:** 10.1007/s00106-021-01050-z

**Published:** 2021-04-14

**Authors:** C. Plettenberg, K. Geipel, I. Stenin, T. Klenzner, M. Wagenmann, J. Schipper, K. Scheckenbach

**Affiliations:** Hals-Nasen-Ohrenklinik, Zentrum für Operative Medizin II, Universitätsklinikum Düsseldorf, Moorenstr. 5, 40225 Düsseldorf, Deutschland

**Keywords:** COVID-19, Pandemie, Tracheotomie, Persönliche Schutzausrüstung, Tröpfcheninfektion, COVID-19, Pandemic, Tracheotomy, Personal protective equipment, Droplet infection

## Abstract

**Hintergrund:**

Die Pandemie COVID-19 („coronavirus disease 2019“) zeichnet sich durch eine hohe Infektiosität bei Tröpfchenübertragung und hoher Virusdichte in den oberen Atemwegen aus. Schwere Krankheitsverläufe stehen mit interstitiellen, beatmungspflichtigen Pneumonien in Verbindung, bei denen regelmäßig Tracheotomien (TT), ein tröpfchen- und aerosolerzeugender medizinischer Eingriff, notwendig werden. Die TT als potenzielles Infektionsrisiko für medizinisches Personal wird in der Literatur kaum behandelt. Deshalb war es Ziel dieser Studie, die Tröpfchenexposition des Op.-Teams während der Tracheotomie zu quantifizieren, um hierdurch die Anforderungen an die notwendige persönliche Schutzausrüstung (PSA) besser zu definieren.

**Material und Methoden:**

Bei 4 nichtinfektiösen Patienten wurde eine chirurgische Tracheotomie durchgeführt, bei der der Chirurg und seine Assistenz jeweils eine chirurgische Mund-Nasen-Maske mit Klarsichtvisier trugen. Nach Durchführung des Eingriffs bestimmten wir Tröpfchenart, -verteilung und -menge auf dem Visier makroskopisch und mikroskopisch.

**Ergebnisse:**

Auf den Visieren fanden sich durchschnittlich 29 Tröpfchen im mittleren Drittel des Visiers, 4 im rechten Drittel und 13 im linken Drittel, bei einer durchschnittlichen Tröpfchengröße von 571 µm (± 381 µm). Die kleinsten Tröpfchen waren 55 µm, die größten 1431 µm groß. Eine Zunahme der Tröpfchen fanden sich bei vermehrter Ventilation während des Eingriffs. Bluttröpfchen waren häufiger als Sekrettröpfchen.

**Schlussfolgerung:**

Es konnte eine deutliche Kontamination des Gesichtsvisiers mit Tröpfchen dargestellt werden. Gerade im Fall einer TT von hochinfektiösen Patienten, z. B. COVID-19, ist demnach die Verwendung einer Kapuzen-Kopfbedeckung in Kombination mit einem Atemschutzgerät mit Luftreinigung mit Stromversorgung empfehlenswert, um einen Infektionsschutz des Operateurs und der Op.-Assistenz bestmöglich zu gewährleisten.

## Abstract (en)

**Background:**

The COVID(coronavirus disease)-19 pandemic is characterized by high infectivity, droplet transmission, and high viral load in the upper respiratory tract. Severe disease courses are associated with interstitial pneumonia and ventilated patients, in whom tracheotomy (TT)—a droplet- and aerosol-producing medical intervention—is regularly necessary. TT as a potential infection risk for medical staff is scarcely found in the literature. Therefore, the aim of this study was to quantify droplet exposure of the surgical team during TT, to better define the requirements for personal protective equipment (PPE).

**Materials and methods:**

Surgical TT was performed in four non-infectious patients, during which the surgeon and his assistant both wore a surgical nasal mask with a transparent visor. After the procedure, the type, distribution, and number of droplets on the visor were determined macroscopically and microscopically.

**Results:**

An average of 29 droplets were found on the middle third of the visor, 4 on the right third, and 13 on the left third, with an average droplet size of 571 µm (± 381 µm). The smallest droplets were 55 µm, the largest 1431 µm. An increase in the number of droplets was found with increased ventilation during the procedure. Blood droplets were more common than secretion droplets.

**Conclusion:**

Contamination of the visor with droplets was demonstrated. Especially in the case of TT in highly infectious patients, e.g., COVID-19 patients, the use of hooded headgear in combination with breathing apparatus with air purification and power supply is recommended to ensure best protection from infection for the surgeon and the surgical assistant.

## Hintergrund

Aufgrund der hohen Infektiosität von COVID-19 („coronavirus disease 2019“) und gleichzeitiger hoher Virusdichte in den oberen Atemwegen müssen Tracheotomien, ein tröpfchen- und aerosolerzeugender medizinischer Eingriff, als potenziell infektiöser Eingriff gelten. Zur Vermeidung von Infektionen und eventuellen schweren Krankheitsverläufen bei medizinischem Personal ist es wichtig, das Infektionsrisiko zu kennen und entsprechende Schutzmaßnahmen auf dieser Grundlage einzuführen.

## Fragestellung

Das SARS-CoV-2-Virus, aus der Gruppe der Coronaviridae, zeichnet sich durch eine hohe Infektiosität und eine Übertragung durch Tröpfcheninfektionen aus. Eine sehr hohe Viruslast wurde im Sekret der oberen Atemwege (Nase/Rachen) detektiert [19]. Zu Risikoeingriffen bei diesen Patienten gehören deshalb Untersuchungen und Eingriffe an den oberen Atemwegen wie Bronchoskopien, Intubationen und Tracheotomien. Eine besondere Exposition von Mitarbeitern im Gesundheitswesen, die vermutlich mit einer erhöhten Rate an Erkrankungen einhergeht, ist im Rahmen der aktuellen Pandemie beschrieben worden [25, 37]. Obwohl die Infektion in den meisten Fällen milde verläuft, gibt es lebensbedrohliche Krankheitsverläufe. Diese zeichnen sich vor allem durch den Übergang der Erkrankung in ein schweres akutes respiratorisches Syndrom (SARS) aus. Die Patienten werden schnell beatmungspflichtig, und im Fall einer Langzeitbeatmung wird eine Tracheotomie zum besseren Atemwegsmanagement, zur Vermeidung von intubationsbedingten Komplikationen und eventuell zur Verbesserung des Outcomes angestrebt. Für den HNO-Arzt steht die Tracheotomie bei COVID-19-Patienten deshalb in einem besonderen Fokus, da es bei der Eröffnung der Trachea regelhaft zu einer Verteilung von Tracheal- und Bronchialflüssigkeiten im Op.-Gebiet und seiner Umgebung kommt. Gleichzeitig findet sich eine besonders hohe Konzentration des Virus in den oberen Atemwegen [39]. Zusätzlich ist bekannt, dass sich SARS-CoV‑2 je nach Oberflächenmaterial und -beschaffenheit bis zu 72 h nachweisen lässt [12]. Bereits bei der SARS-Epidemie 2004 zeigte sich, dass insbesondere das medizinische Personal durch seine Patientenexposition gefährdet ist. So waren z. B. in Hongkong 400 von 1755 SARS-Patienten Beschäftigte im Gesundheitswesen [3]. Sorgfältige Beachtung der Infektionskontrolle und adäquater Infektionsschutz sind deshalb unerlässlich, um Übertragungen des Virus vom Patienten auf Mitarbeiter zu minimieren. Die Dringlichkeit zur Prävention im Vergleich zu anderen Viren ergibt sich aufgrund der hohen Infektiosität und der hohen Transmissionsraten [16]. Um einen Eindruck über die Tröpfchenexposition, -verteilung und -art zu bekommen, wurden bei nichtinfektiösen Patienten die Visiere des verwendeten Mund-Nasen-Schutzes mit integriertem Visier untersucht.

## Material und Methoden

### Operatives Setting und Asservation der Schutzmasken

**Figure 1:**
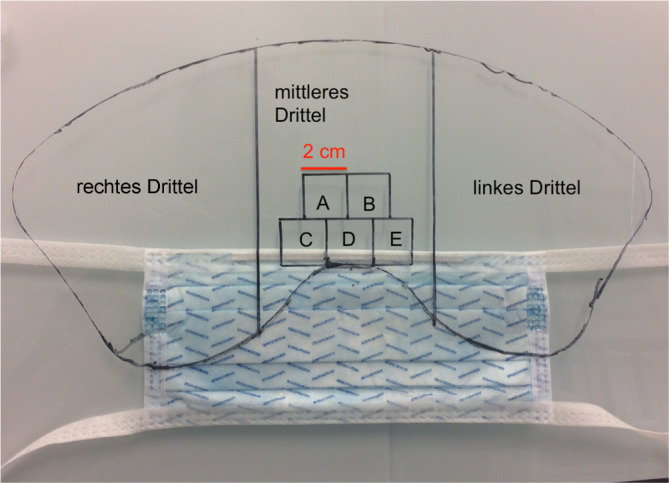

Es wurden 4 langzeitbeatmete nichtinfektiöse Patienten chirurgisch von je 2 Operateuren tracheotomiert. Alle Beteiligten hatten einen Abstand von 30–80 cm vom Operationsgebiet und waren der direkten perioperativen Umgebung ausgesetzt. Während der Prozedur trugen die beiden Operateure und die Assistenz eine Mund-Nasen-Maske mit integriertem Gesichtsschutz (Sentinex Op.-Maske Fluid Shield der Fa. Lohmann und Rauscher [Neuwied, Deutschland]; Abb. 1). Diese (*n* = 8) wurden nach Beendigung der Operation ohne Oberflächenkontakt bei Erhalt der postoperativen Oberflächenbeschaffenheit anonym asserviert, für mindestens 24 h getrocknet und an diesem Material unter Durchleuchtung eine Analyse von Tröpfchenanzahl und -beschaffenheit durchgeführt.

Zur Analyse wurden die Masken in 3 Teilbereiche (frontal, lateral rechts, lateral links) mit je gleicher Breite aufgeteilt (Abb. 1). In Rücksprache mit der hiesigen Ethikkommission ist für die patientenunabhängige Materialanalyse der Masken keine Einwilligung des Patienten notwendig. Alle beschriebenen Untersuchungen wurden in Kooperation mit der zuständigen Ethikkommission, im Einklang mit nationalem Recht sowie gemäß der Deklaration von Helsinki von 1975 (in der aktuellen, überarbeiteten Fassung) durchgeführt.

### Messung der Tröpfchendichte und -qualität

**Figure 2:**
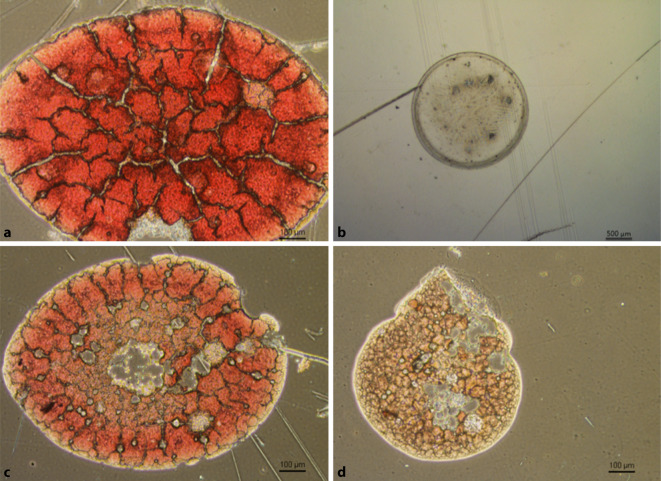

Es erfolgte die makroskopische Auszählung der gesamten Maskenfläche. Der Bereich oberhalb der Nase wurde in 5 Areale von je 2 × 2 cm Größe eingeteilt und zusätzlich mikroskopisch in 2,6facher Vergrößerung ausgezählt. In einem repräsentativen Areal, 20 cm^2^ im zentralen Bereich um Nase und Auge jeder Maske, wurde in 4‑ und in 10facher Vergrößerung mikroskopisch die Zusammensetzung der Tröpfchen (Sekret/Blut) und die Größe der Tröpfchen analysiert. Hierbei wurden je Feld die Tröpfchen anhand einer im Mikroskop integrierten Skala ausgemessen. Außerdem erfolgte eine Fotodokumentation der Befunde (Abb. 2).

#### Statistische Auswertung

Für die Tröpfchenverteilung und -größe wurden Mittelwerte und Standardabweichungen sowie die Odds Ratio arealbezogen berechnet.

## Ergebnisse

### Auswertung der Tracheotomien

Bei insgesamt 4 Tracheotomien konnten 8 Gesichtsschilde ausgewertet werden. Es liefen 3 Eingriffe regelrecht ohne Komplikationen ab. Bei einem Eingriff kam es intraoperativ durch Schlitzung mit dem Skalpell zu einem Defekt des Tubus-Cuffs ohne weitere negative Auswirkungen auf den Operationsverlauf. Die Visiere des Eingriffs wurden jedoch zusätzlich gesondert betrachtet.

### Auswertung der Tröpfchengröße und -verteilung

**Figure 3:**
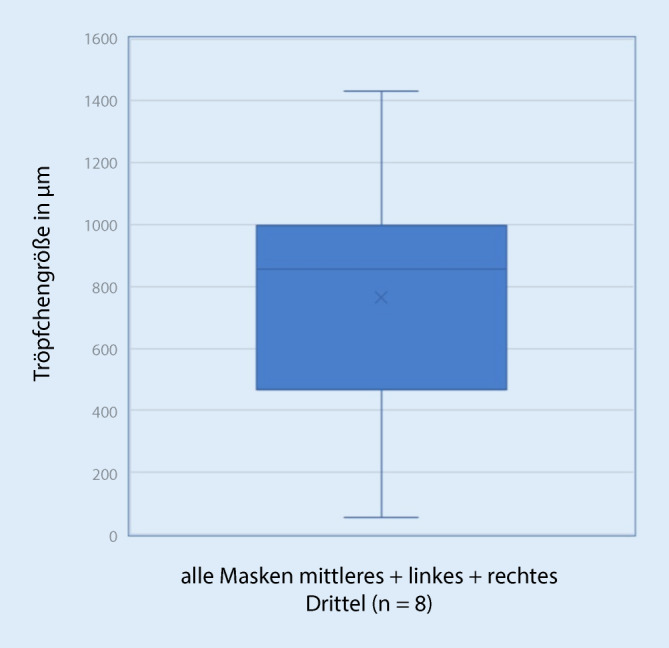

**Figure 4:**
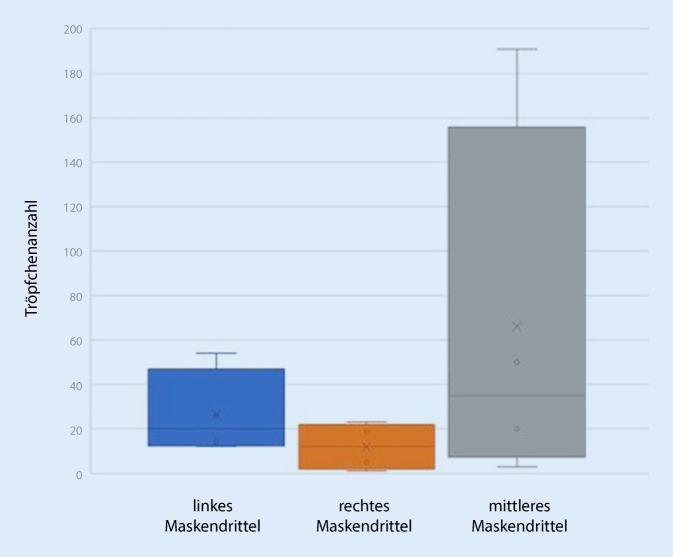

**Figure 5:**
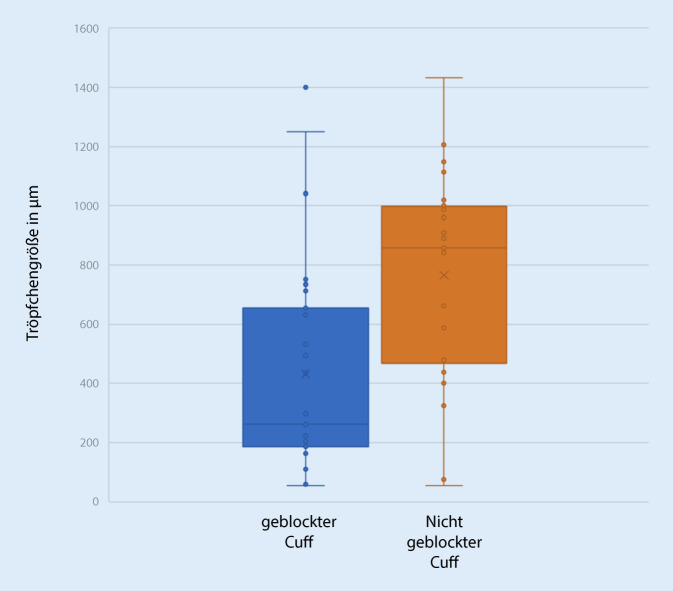

In jedem der 4 Eingriffe kam es zur Tröpfchenübertragung auf das Visier. Die Analyse der Gesichtsschilde ergab Tröpfchengrößen zwischen 55 und 1431 µm (Tab. 1; Abb. 3), mit einem Mittelwert von 571 µm bei einer Standardabweichung von 381 µm. Die mittlere Anzahl der Tröpfchen betrug im mittleren Drittel des Gesichtsschilds 29,13 Tröpfchen (± 37,15 Tröpfchen), im rechten Drittel 4,38 Tröpfchen (± 5,70 Tröpfchen), im linken Drittel 13,25 Tröpfchen (± 9,34 Tröpfchen; Tab. 2; Abb. 4). Eine Exposition bestand demnach vor allem erwartungsgemäß im mittleren Drittel. Die Expositionswahrscheinlichkeit im mittleren Drittel war demnach 1,65-mal höher als in der Peripherie (Odds Ratio 1,65). Mikroskopisch wurden insgesamt 63 repräsentative Tröpfchen in 4facher und 10facher Vergrößerung ausgewertet. Hierbei fanden sich Tröpfchen mit vermehrten Anteilen von Blut, bei der die zellulären Blutbestandteile, in erster Linie Erythrozyten, gut zur Darstellung kamen (Abb. 2a), außerdem zeigten sich Tröpfchen ohne zelluläre Bestandteile, die dem austretenden Sekret entsprachen (Abb. 2b), sowie Tröpfchen mit gemischten Anteilen (Abb. 2c, d). Das Gesamtverhältnis der Bluttröpfchen zu Sekrettröpfchen war 12,7:1. Bluttröpfchen ließen sich auf allen 8 Shields nachweisen, gemischte oder reine Trachealsekrettropfen nur auf 6 von 8. Trachealsekrettröpfchen fanden sich 1,8-mal häufiger in der Peripherie als in der Mitte, wobei auch in der Mitte die Verteilung außerhalb des Zentrums war. Bei dem Patienten mit dem defekten Cuff und der Zwischenbeatmung war die Tröpfchenzahl mit 265 auf dem gesamten Shield deutlich höher als bei den anderen Patienten (Mittelwert 50/Shield). Die Tröpfchengröße bei dem Patienten mit defektem Cuff war kleiner als bei den anderen Patienten, Mittelwert 431 ± 261 µm zu 765 ± 340 µm, Median 261 µm zu 857 µm (Abb. 5). In allen Eingriffen kam es vor allem im zentralen Drittel der Maske zu einer Tröpfchenexposition (Tab. 2).

**Table 1:** 

Minimale Tröpfchengröße	55 µm
Maximale Tröpfchengröße	1431 µm
Mittlere Tröpfchengröße (± Standardabweichung)	571 µm (± 381 µm)

**Table 2:** 

	Gesamtes Visier	Linkes Drittel	Rechtes Drittel	Mitte	20 cm^2^	Mittlere Tröpfchenanzahl aller Visiere (TT1–4)
	(*n* = 2)	(*n* = 2)	(*n* = 2)	(*n* = 2)		
*Tröpfchen Blut und Sputum*						
TT1	268	54	23	191	54	105
TT2	37	12	5	20	4	
TT3	18	14	1	3	3	
TT4	95	26	19	50	18	
*Tröpfchen Blut*						
TT1	260	51	20	189	44	98
TT2	24	7	5	12	2	
TT3	18	14	1	3	3	
TT4	90	21	19	50	18	
*Tröpfchen Sekret*						
TT1	8	3	3	2	0	7
TT2	15	7	0	8	1	
TT3	0	0	0	0	0	
TT4	5	5	0	0	0

## Diskussion

Die Infektionsübertragung durch Tröpfcheninfektionen im Verlauf einer Tracheotomie bekommt im Zusammenhang mit der neuartigen Erkrankung COVID-19 einen ganz neuen Stellenwert. Der SARS-CoV-2-Erreger ist nicht nur hochinfektiös [20], er ruft teils schwere, lebensbedrohliche Krankheitsverläufe hervor, bei denen in erster Linie eine interstitielle Pneumonie mit mangelnder Ventilation zum Tod des Patienten führen kann. Bei limitierten und primär symptomatischen Therapiemöglichkeiten steht hier die suffiziente Beatmung des Patienten im Vordergrund. Langzeitbeatmete Patienten benötigen sehr häufig eine Tracheotomie. Diese reduziert Komplikationen durch den endotrachealen Tubus (laryngeale und tracheale Druckschäden) [4, 26, 29], verkürzt die Beatmungszeit und verringert das Infektionsrisiko der unteren Atemwege [27]. Außerdem werden das Totraumvolumen, der Atemwiderstand, die Atemarbeit [3, 8, 10, 32, 33, 38] und der Sedierungsbedarf reduziert und Mobilisations- und Kommunikationsmöglichkeiten verbessert [33]. In der AWMF-S1-Leitlinie „Empfehlungen zur intensivmedizinischen Therapie von Patienten mit COVID-19“ sind die Vor- und Nachteile einer Tracheotomie, Wahl des Tracheotomiezeitpunkts und der Tracheotomieart zusammengefasst [17].

Aerogene Infektionen, wie SARS-CoV‑2, aber auch SARS-CoV, MERS-CoV („Middle East respiratory syndrome-related coronavirus“) oder Influenzaviren, erfolgen über Aerosole (≤5 μm – Übertragungsweg > 1 m) und Tröpfchen (>5 μm – Übertragungsweg < 1 m) [5, 8, 14, 30]. Diese werden durch Atmen, Husten, Niesen, Sprechen, Lachen, aber auch bei aerosol- und tröpfchenproduzierenden Untersuchungen oder Eingriffen übertragen. Tröpfchen sedimentieren innerhalb weniger Sekunden, wodurch Tröpfchenkerne entstehen, die als Aerosole in der Luft schweben können [2].

Zu den Prozeduren im Gesundheitswesen, die Aerosole und Tröpfchen in hohem Maße erzeugen, gehören Bronchoskopien, das Absaugen der Atemwege und die Intubation [5]. Informationen zur Tröpfchenbildung während der Tracheotomie sind bisher rar, obwohl diese perioperativ offensichtlich sind. Thamboo et al. [35] fassten die aktuelle Literatur zur Eingriffen mit Aerosol- oder Tröpfchenbildung zusammen. Als tröpfchenerzeugendes Verfahren erhöht die Tracheotomie die potenzielle Virusexposition gegenüber den beteiligten Personen [15, 16, 28, 35]. Wir konnten eine starke Tröpfchenbildung und somit Exposition des Operateurs anhand von Untersuchungen der Visiere der Operateure zeigen. Die Tröpfchengrößen variierten in ihrer Größe stark, sind aber eher als großvolumig anzusehen. Eine verhältnismäßig hohe Virusmenge ist deshalb im Fall von COVID-19-Patienten in den Tröpfchen wahrscheinlich. Hierfür spricht auch, dass neben Blutbestandteilen oftmals wesentliche Sekretanteile in den Tröpfchen mikroskopisch darstellbar waren. Eine besondere Tröpfchenexposition liegt, wie erwartet, im gesamten frontalen Gesichtsbereich mit einem Schwerpunkt im Nasenrücken‑/Augenbereich vor. Doch auch laterale Gesichts- und Schädelbereiche waren deutlich exponiert. Da alle Messungen oberhalb der Nasolabialfalte erfolgten, kann nur angenommen werden, dass auch der Mundbereich betroffen ist. Die persönliche Schutzausrüstung (PSA) kann, aus Sicht der Autoren, insofern nicht durch einfache Schutzbrillen in Kombination mit Halbmasken gewährleistet werden, sondern sollte durch eine frontale und laterale Ummantelung des Gesichts sichergestellt werden. Auch der Mund- und Nasenschutz sollte eine möglichst dichte und vollständige Ummantelung gewährleisten, da die Gefahr der Tröpfchenexposition bei nicht richtig anliegender Maske in die Atemwege des Operateurs ansonsten zu befürchten ist. Eine ausreichend sichere PSA zur Minimierung des Infektionsrisikos ist gerade im Umgang mit COVID-19-Patienten unumgänglich [18, 22]. Auf Grundlage dieser Berichte und der Veröffentlichung einer aktuellen multidisziplinären Empfehlung von McGrath et al. [23] sollte eine PSA als Mindestanforderung aus einem FFP3-/N95-Mund-Nasen-Schutz, einem Schutz der Augen, einem flüssigkeitsabweisenden Einweg-Operationskittel und Handschuhen bestehen [30, 31]. Dabei ist nicht nur das Tragen der PSA, sondern auch das korrekte Anlegen und Ablegen der PSA ein wichtiger Faktor in der Vermeidung von Infektionen [15, 16, 34, 37].

Die Empfehlungen der Fachgesellschaften [1, 9, 13, 17], die auf der Grundlage von Beobachtungen der aktuellen COVID-19-Pandemie, aber auch der SARS-Pandemie von 2003 und 2004 sowie der MERS-Pandemie von 2007 entstanden sind, sehen hier in der Regel FFP2- oder FFP3-Masken vor, ggf. zusätzlich Schutzbrillen [3]. Die Klassifikation der verfügbaren filtrierenden Halbmasken („filtering face piece“, FFP) wird nach europäischen Normen (DIN EN 149, Tab. 3) vorgenommen [11]. Sie beschreibt die Gesamtleckage der Maske an den Undichtigkeitsstellen, die sich durch die Undichtigkeiten am Gesicht und Ausatemventil ergeben.

**Table 3:** 

Maske	Gesamtleckage (%)	Mittlerer Partikeldurchmesser	Schutz vor
FFP1	≤ 25	0,6 µm	Tröpfchen/Aerosolen aus der Umgebung
FFP2	≤ 11	0,6 µm	Tröpfchen/Aerosolen aus der Umgebung
FFP3	≤ 5	0,6 µm	Tröpfchen/Aerosolen aus der Umgebung
Mund-Nasen-Schutz	≥ 25	–	Abgabe von Tröpfchen durch den Träger

Das Tragen von Op.-Masken mit Gesichtsschild oder von Schutzbrillen schützt zusätzlich vor transokulären Infektionen durch Tröpfchen. Bei mutmaßlichem Risiko der transokulären Übertragung [40] ist demnach ein okulärer Schutz im Umgang mit COVID-19-Patienten essenziell. Sowohl die Verwendung von Gesichtsschilden als auch Schutzbrillen in Kombination mit Halbgesichtsmasken garantieren jedoch keinen umfassenden Schutz des Gesichts und seitlichen Kopfs. Die Tröpfchenexpositionsanalysen unserer Gesichtsschilde stellen jedoch auch in lateralen Gesichtsanteilen und der betrachteten Gesichtsfläche eine hohe Tröpfchenexposition dar, sodass auch eine Tröpfchenverteilung darüber hinaus denkbar ist. FFP2- und FFP3-Halbmasken in Kombination mit einfachem okulärem Schutz bieten deshalb, aus Sicht der Autoren, einen lückenhaften Schutz und sind demnach für die Durchführung von Tracheotomien kein ausreichender Personenschutz bei hochinfektiösen Erkrankungen, die durch Tröpfchen übertragen werden. Die Einhaltung des kürzlich beschriebenen Verfahrens zur Tracheotomie mit Pausieren der Beatmung im Moment der Trachealeröffnung [28] reduziert die Tröpfchenexposition erheblich. Bei den in dieser Arbeit beschriebenen 3 Tracheotomien ohne Cuffruptur kam es ebenfalls, wenn auch in einem deutlich geringeren Ausmaß, zu einer Tröpfchenexposition der Operateure.

Bei der SARS-Epidemie 2004 zeigte sich, dass insbesondere das medizinische Personal durch seine Patientenexposition gefährdet ist [18]. In Studien von Loeb et al. [21] und MacIntvre et al. [24] zeigte sich eine gleich hohe Infektionsrate des medizinischen Personals während einer SARS-Epidemie unabhängig davon, ob eine FFP2-Maske oder ein chirurgischer Mund-Nasen-Schutz verwendet wurde. Die hohen Infektionsraten und der fehlende Nachweis des Vorteils von FFP2-Masken kann auf eine fehlerhafte Verwendung der Atemschutzmasken, transokuläre Infektion trotz angemessener Verwendung von Atemschutzmasken, ohne Augenschutz oder Infektionen im privaten Umkreis zurückzuführen sein [27]. Es kann aber auch die unzureichende seitliche Abschirmung mit ursächlich sein. Canelli et al. berichteten über eine definierte Messung der Tröpfchenexposition bei endotrachealer Intubation [6]. Hier wurde mithilfe einer „Aerosol Box“ und fluoreszenzgefärbtem artifiziellem Sekret ein Hustenstoß bei Intubation simuliert und die Tröpfchenausbreitung dargestellt. Tröpfchen waren auf dem Kittel, den Handschuhen, der Gesichtsmaske, dem „face shield“, aber auch an Haaren, Hals, Ohren und Schuhen des Untersuchers nachweisbar. Außerdem kamen sie auf dem Boden in 1 m Entfernung und auf einem 2 m entfernten Monitor zur Darstellung. Die Tracheotomie bietet, gerade bei abrupt defektem Cuff, als Komplikation eines chirurgischen Eingriffs, eine vergleichbare Tröpfchenexposition zur Intubation. Erhöhte Sicherheitsmaßnahmen mit adäquatem Schutz des Personals sind demnach erforderlich [27]. Deshalb müssen auch die interdisziplinären Abläufe, wie An- und Ablegen der PSA, das Vorschieben des Tubus carinanah, das Stoppen der Beatmung während der Tracheaeröffnung und der Start der Beatmung gut kommuniziert und im Vorfeld trainiert werden. Pudszuhn et al. berichten auf der Grundlage eines standardisierten interdisziplinären und im Vorfeld trainierter Ablaufs (An- und Ablegen der PSA unter Buddy-Check, Vorschieben des Tubus carinanah, Pausieren der Beatmung kurz vor der Trachealeröffnung, gut kommunizierter Start der Beatmung), dass dieses Vorgehen zur Vermeidung der Bildung von Aerosolen und Tröpfchen beitragen kann [7, 28]. Dabei verweisen sie besonders auf die Wichtigkeit des Vorschiebens des Cuffs carinanah und auf das Sistieren der Beatmung während der Trachealeröffnung zur Vermeidung von Aerosolen und Tröpfchenbildung.

Kempfle et al. [16] beschreiben und analysieren das Management tracheotomierter Patienten. Dabei diskutieren die Autoren auch den umfassenden Schutz eines PAPR in Kombination mit einer Vollgesichts-Atemschutzmaske, die bei COVID-Tracheotomien in unserer Klinik auch zum Einsatz kommen. PAPR bestehen in der Regel aus einer Vollgesichtsmaske oder Haube, einer batteriebetriebenen Luftpumpe und einem Hochleistungsfilter. Bei diesen leistungsstarken luftreinigenden Atemschutzgeräten (PAPR) wird die Umgebungsluft vor dem Einatmen durch einen hocheffizienten HEPA-Filter („high-efficiency particulate air“) geführt. Hierdurch wird die Filtrationsleistung gegenüber FFP-Atemschutzgeräten erhöht [22, 36], dies ist wichtig, da auch FFP3-Masken ein Aerosolinhalationsrisiko nicht vollständig ausschließen [16, 36]. Die PAPR umhüllt zumeist den gesamten Gesichtsschädel, schließt unter dem Op.-Mantel ab und bieten somit keine Eintrittspforte für die erregerhaltigen Tröpfchen [36]. Ein weiterer Vorteil ist, dass die Luftableitung dorsal, also patientenabgewandt erfolgt. Sie weisen darauf hin, dass je nach Modell ungereinigte Luft des Operateurs ein theoretisches Kontaminationsrisiko des sterilen Operationsfelds darstellen kann [16, 36], schränken dies allerdings modellbezogen ein und verweisen hierbei auf die zu diesem Thema nur begrenzten Daten hin. Die Vor- und Nachteile von PAPR, aber auch von anderen Maskentypen wurden im Rahmen der Influenza-Pandemie 2009 (H1N1) durch Tompkins und Kerchberger [36] umfassend dargestellt. Bei der Betrachtung der Vorzüge der PAPR legen sie für tröpfchen- und aerosolerzeugende Eingriffe die Verwendung eines „powered air-purifying respirator“ (PAPR) nahe. Givi et al. ergänzen dies in Bezug auf die aktuelle Pandemie und weisen auf die Schwierigkeiten beim An- und Ausziehen und die damit verbundene Infektionsgefahr des medizinischen Personals, die Lautstärke der PAPR und damit verbundene eventuelle Verständnisschwierigkeiten explizit hin [32]. Alle Arbeiten verweisen ebenfalls auf die hohen Kosten und fehlende vergleichende Studien zu Infektionen von medizinischen Personal zwischen FFP3-/N95-Masken und Schutzbrille/Gesichtsschild. PAPR sind in den nationalen und internationalen Leitlinien und Empfehlungen zur Tracheotomie [1, 9, 12] nicht explizit empfohlen, ihr hoher Infektionsschutz wird aber herausgestellt.

Unsere Pilotstudie unterstützt die Empfehlungen der nationalen und internationalen HNO-Fachgesellschaften (Deutsche Gesellschaft für Hals-Nasen-Ohren-Heilkunde, Kopf- und Hals-Chirurgie e.V., DGHNO-KHC; American Academy of Otolaryngology–Head and Neck Surgery, AAO-HNS; Ear, Nose and Throat surgery United Kingdom, ENT-UK) im Umgang mit SARS-CoV‑2 [1, 9, 12]. Hiernach werden übereinstimmend bei Vorliegen oder Verdacht auf eine COVID-19-Erkrankung mindestens eine FFP2-Schutzmaske in Kombination mit einem okulären Schutz bei Untersuchungen und Operationen der oberen Atemwege sowie ein erweiterter Schutz bei besonderer Exposition, wie er bei der Durchführung einer Tracheotomie vorliegt, empfohlen.

## Fazit für die Praxis

In dieser Studie wurde eine ausgeprägte Tröpfchenexposition des medizinischen Personals bei Tracheotomien aufgezeigt. Bei infektiösen Patienten, aber insbesondere bei COVID-19-Patienten, sollten maximale Ansprüche an die persönliche Schutzausrüstung gestellt werden.
